# Diagnostic Delay in Coeliac Disease: A Survey among Danish Patients

**DOI:** 10.1155/2022/5997624

**Published:** 2022-12-28

**Authors:** Line Lund Kårhus, Susanne Hansen, Jüri J. Rumessen, Allan Linneberg

**Affiliations:** ^1^Center for Clinical Research and Prevention, Copenhagen University Hospital-Bispebjerg and Frederiksberg, Copenhagen, Denmark; ^2^Department of Clinical Medicine, Faculty of Health and Medical Sciences, University of Copenhagen, Copenhagen, Denmark

## Abstract

**Background:**

Coeliac disease affects around 1% of the population, although many cases remain undiagnosed. Underdiagnosis and diagnostic delay in coeliac disease may cause health complications and be a burden for both the patient and society. Casuistic reports indicate that the diagnostic delay may be significant in Danish patients.

**Aim:**

To investigate the diagnostic delay among Danish patients with coeliac disease.

**Methods:**

We performed a survey among coeliac disease patients to investigate the diagnostic delay. A web-based questionnaire was sent to all members of The Danish Coeliac Society.

**Results:**

The questionnaire was completed by 1,392 individuals with a diagnosis of coeliac disease (78.1% women; mean age: 42.8 years). The mean delay was 1.8 (SD 5.0) years from the first symptom to the first health care contact and 5.8 (SD 9.5) years from the first symptom to diagnosis; 18.6% of the participants reported a total diagnostic delay of more than 10 years. Among the patient-reported reasons for delay were misunderstandings, unspecific symptoms, and a lack of knowledge or focus on coeliac disease among the doctors. In total, 52.7% rated the time to diagnosis to have been “too long,” and 20.1% were not satisfied with the diagnostic process. However, the majority were “to some extent” or “very” satisfied with the diagnostic process.

**Conclusion:**

We found evidence of a significant diagnostic delay among Danish patients with coeliac disease. This was primarily due to the delay from the time of first health care contact to the time of diagnosis. This study highlights the importance of raising awareness of coeliac disease among health care professionals.

## 1. Introduction

Coeliac disease is a chronic autoimmune disease caused by an abnormal immune response triggered by the ingestion of gluten-containing grains (wheat, rye, and barley) in genetically susceptible individuals [1]. Coeliac disease is a systemic disease occurring at every age, affecting around 1% of the population [2]. However, many cases of coeliac disease remain undiagnosed [1, 3].

There are several reasons for the underdiagnosis of coeliac disease, but the diverse clinical presentation, and in many cases, the absence of symptoms have been found to affect a large proportion of underdiagnosed individuals with coeliac disease. The broad clinical picture can also cause a diagnostic delay, e.g., prolonged time from the onset of the first symptoms until the time of diagnosis. In some studies, the average diagnostic delay has been long, but there is a wide variation between the reported mean delays, from a few years up to over 10 years [4–9]. The long diagnostic delay can be a potential burden both for the patient and society, resulting in more health-care contacts and expenses, sick-leaves, etc. [10–13], as well as slower improvement after the start of treatment and an increased risk of health complications [5, 7, 8]. The diagnostic delay can be divided into patient delay, referring to the duration of symptoms prior to the first doctor visit, and doctor delay referring to the time from the first doctor visit until the time of diagnosis. In Denmark, health-care services are provided free of charge, and all Danish citizens have an allocated general practitioner (GP). The GPs are the gatekeepers in the Danish health-care system, and a referral is needed for visits in secondary health care and to specialists in private practice in other specialties than general practice.

Casuistic reports indicate that the diagnostic delay may also be significant in Denmark [14], although representative surveys investigating the time from the first symptom/health care contact to the diagnosis of coeliac disease have, to our knowledge, not previously been performed in Denmark. We therefore aimed to investigate the diagnostic process and possible delays in coeliac disease by performing a questionnaire-based survey among Danish coeliac disease patients.

## 2. Methods

The present study was performed in collaboration with The Danish Coeliac Society (DCS), the patient organization for patients with coeliac disease in Denmark. The questionnaire was developed in collaboration with two representatives from DCS. The board of DCS, including patients, was invited to propose questions and topics, and they approved the final questionnaire. The questionnaire included questions on symptoms, with both prespecified symptoms with tick boxes and free-text as answer possibilities, the diagnostic process and onset of symptoms, the first health-care visit, and the date of diagnosis. Furthermore, it included questions on health, follow-up, and health-care control visits. The survey was sent out by e-mail, along with a link to the web-based questionnaire to all members of the DCS. On May 5, 2021, DCS had 2,978 members (68.9% women) including coeliac disease patients and family members. Of the 2,978 memberships, 209 were family memberships, where several family members are linked to the same membership. Therefore, the questionnaire both had the possibility of a closed link directly to the member's e-mail and an open link to other coeliac disease patients for the possibility of several respondents, e.g., family members diagnosed with coeliac disease. The data collection was conducted by Epinion (https://epinionglobal.com/) during April and May 2021.

The data from the individual questionnaires were transferred to a statistical software package, SAS Enterprise Guide 7.1 (SAS Institute, NC, USA), and descriptive statistics were performed with calculations of frequencies and proportions (*n* and %) and means with standard deviations (SD). For the calculation of diagnostic delay, only participants with information on dates of symptom onset, first doctors' visit and diagnosis of coeliac disease were included in the delay study population. For participants with dates with information on year, but with missing information on month (months recorded as “unknown”), the month was set to June. However, if the participant had other dates from the same year (symptom onset, first doctor's visit, or diagnosis) recorded with month, then the month was set as the same month as the recorded date.

According to Danish regulations, questionnaire surveys do not require ethical approval.

## 3. Results

In total, 1,399 individuals answered the questionnaire, but seven were excluded due to a lack of diagnosis of coeliac disease, resulting in a study population of 1,392 individuals with coeliac disease. However, due to missing data on dates, 153 individuals were excluded for the analyses of diagnostic delay, which therefore included 1,239 individuals.

The participants were between 3 and 87 years old, the mean age: 42.7 years, and 78% were women (Table 1). Most patients had been diagnosed with coeliac disease at a hospital (77%), while 11% had been diagnosed by their general practitioner and 12% by a specialist in private practice. The patients were requested to report reasons for their diagnosis, and the majority reported symptoms (63%) and/or health problems (59%), while 12% were diagnosed due to coeliac disease in the family.

The participants were asked what they thought about the duration from the first doctor visit until diagnosis; 53% (733/1,392) answered that the time was “way too long” or “a little too long,” 21% (299/1,392) thought the duration was appropriate, and 17% (230/1,392) answered that it went fast, while 130/1,392 (9%) did not know or did not remember. Participants were also asked about the reason for the delay from the first doctor's visit until the diagnosis of coeliac disease. The most frequent answers were that they were not taken seriously, that they underwent examinations for other conditions due to a lack of symptoms, misunderstandings, that the doctor did not think it was coeliac disease or did not think about it, and that they had to wait in the health care system. The self-perceived health of the participants, the percentage of controls during the last 12 months, and limitations on daily life is shown in Table 2. The majority stated that they were to some point limited, and when asked what situations they were limited by their disease, 93% (1,288/1,392) answered when eating out and/or attending social events, 74% (1,035/1,392) felt restricted when traveling, 52% (729/1,392) at work/school/kindergarten, 32% (448/1,392) were limited during spare time activities, and 10% (143/1,392) felt they were limited when eating at home. The distribution of the self-reported symptoms in this study population is shown in Table 3. Only 2% reported not having had any symptoms.

**Figure 1:**
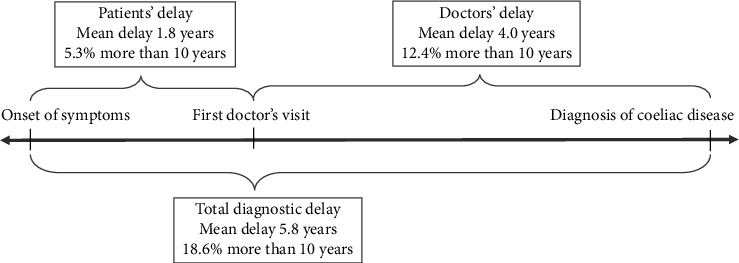
Self-reported diagnostic delay in a Danish population of patients with coeliac disease.

Figure 1 shows the diagnostic delay for the 1,239 participants with data available for these analyses. The patients' delay, e.g., the time from the first symptom until the first doctor's visit, showed a mean of 1.8 years (SD 5.0). 5.3% had a patients' delay of more than 10 years; and 14% had a patients' delay of more than 3 years. The time from the first doctors visit until the diagnosis, the doctors' delay, had a mean duration of 4.0 years (SD 8.0); 12.4% had a doctors' delay of more than 10 years; and 26% had a doctors' delay of more than 3 years. Lastly, the total diagnostic delay, time from the first symptom until a diagnosis of coeliac disease showed a mean total diagnostic delay of 5.8 years (SD 9.5), 18.6% had a total diagnostic delay of more than 10 years, and 38% had a total diagnostic delay of more than 3 years (Table 4). Among the 1,200 participants in the delay study population with data on age, the mean age at diagnosis was 32.5 years.

The diagnostic process changed over time, and Table 4 shows the changes in diagnostic delay over the different time periods and illustrates a decrease in delay during the recent two decades. The diagnostic process also changes with age at diagnosis, and Table 4 also shows the changes in diagnostic delay by age at diagnosis for diagnoses given between year 2000 and 2021, showing that adults between 40 and 59 years of age have the highest mean years of delay before the diagnosis of coeliac disease is reached.

**Figure 2:**
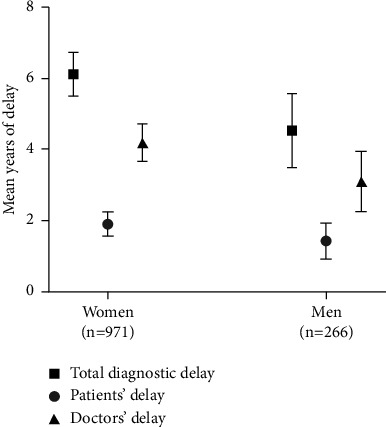
Self-reported diagnostic delay in a Danish population of women and men with coeliac disease. Diagnostic delay reported in mean years of delay with 95% confidence intervals.

This study also found that women had a longer diagnostic delay than men; a mean total diagnostic delay of 6.1 (95% confidence interval (CI) 5.5–6.7) years among women vs. 4.5 (95% CI 3.5–5.6) years in men (Figure 2).

We compared the frequency of symptoms reported by participants with a diagnostic delay of more than 10 years with the frequency of symptoms reported by participants with a diagnostic delay of less than 10 years. The group with the long delay reported markedly more often anaemia, joint pain, infertility, and osteoporosis (Supplemental online material, Table S1).

## 4. Discussion

**Figure 3:**
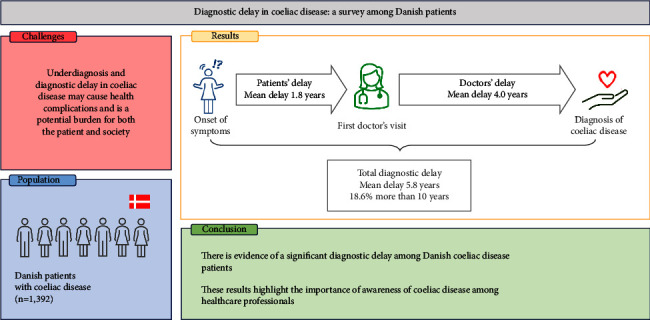
Graphical illustration of the study.

This study showed that Danish coeliac disease patients often experience a significant diagnostic delay; both from the first symptom and from the first health care contact to the diagnosis of coeliac disease (Figure 3).

To our knowledge, this is the first survey-based study performed in Denmark, but a clinical study from 1995 reviewed medical records from 50 coeliac disease patients at a specific hospital department and found a median diagnostic delay of 3 years [15]. However, diagnostic delay in coeliac disease has been assessed in studies performed in other countries [4–9]. The reported results differ considerably, and our results show a lower diagnostic delay than the long delay of 10–13 years reported in some studies [6, 9, 16, 17] but are more comparable to those of Häuser et al. [18]. Moreover, Vavricka et al. [5] found similar findings to ours with respect to doctors' mean delay of 3.2 years; however, we found a lower patients' delay than the mean of 3.4 years found in the study from Switzerland. This study also showed a sex difference driven by the doctors' delay, and we found a comparable sex difference and a higher mean doctors' delay for women than for men.

Some studies have observed a decrease in diagnostic delay over time [6, 7, 17]. In our study, we found that the mean delay was lower among the diagnoses made during year 2000–2021 compared with 1980–1999 regarding the total and doctors' delay, but the patients' delay was not markedly different from the early period with a mean patient delay of 1.9 (5.6) years to the later period with mean patient delay of 1.8 (5.0) years. However, it is important to note that the majority of participants in this study were diagnosed during the years 2000–2021, a fact that could hamper an accurate assessment of time trends in diagnostic delay.

In comparison to Fuchs et al. [7], who found that 32% had a total diagnostic delay of more than 10 years, we found a lower percentage of total diagnostic delay over 10 years (18.6%). We found that 38% had a total diagnostic delay of more than 3 years, comparable with numbers from Tan et al. [8] who found that 40% had a total delay over 3 years. Tan et al. [8], further found that a delay over 3 years was associated with slower improvement of symptoms after diagnosis and initiation of treatment, highlighting the importance of improved diagnosis of coeliac disease to reduce the burden of the disease. We did not have access to clinical data in our study, but investigations into the consequences of diagnostic delay and untreated coeliac disease would be an important focus in further studies. However, we did have information on symptoms and found that both participants with and without a delay over 10 years reported many symptoms, although the group with a long delay had slightly higher percentages of all symptoms except failure to thrive and weight loss. It is important to note that a limitation of the survey is that we did not have clinical information or laboratory results from patient records before or at the time of diagnosis. This would be relevant since other more specific clinical/laboratory features could lead to a diagnosis of coeliac disease, and patients with coeliac disease often present with biochemical abnormalities or other clinical presentations, e.g., biochemical hypertransaminasemia [19] or neurological disorders [20, 21]. Furthermore, this study population is a population of people with diagnosed coeliac disease, and other factors leading to an even longer delay or no diagnosis cannot be investigated in this study. However, the long diagnostic delay and slightly higher percentages of symptoms found in this study, as well as the clinical experience of several comorbidities in coeliac disease, such as iron-deficiency anaemia and autoimmune disorders, show the importance of serological screening for coeliac disease in patients with biochemical abnormalities [22], unspecific symptoms, or other disorders known to be associated with coeliac disease, to avoid diagnostic delay and underdiagnosis.

We found a lower degree of satisfaction among the patients than Green et al. [9]; they found that in a US population, 53% considered their diagnosis to be prompt, but only 21% in our study found the time to diagnosis suitable, and 40% were not at all or only a little satisfied with the diagnostic process. There are, however, large differences between the Danish and US health care systems, which limit the comparability of results.

Our study both included adults and children, although mostly adults; when comparing the participants under the age of 18 years at diagnosis with a study in children, we found a higher rate of delay than Riznik et al. [23]. They found 6.6% of children to have a diagnostic delay over 3 years, while we found a total diagnostic delay over 3 years of 25% among participants under the age of 18 years at diagnosis. Furthermore, our results are not in line with an Italian study [24], who found that the diagnostic delay was higher in elderly over 65 years of age, as we found the highest mean diagnostic delay among the participants aged 40–59 years. These differences could be due to the selection of populations, as Riznik et al. [23] and Gasbarrini et al. [24] included patients through paediatric gastroenterologists and gastroenterology units, respectively.

The possibility of a broader picture of the diagnostic delay in Denmark, in all geographical regions, and in all ages is a strength of our study. However, the selection of the study population being members of the Danish Coeliac Association could decrease the representativeness of the results. Recall bias might also be a limitation, and it may be hypothesized that patients diagnosed decades ago tend to underestimate diagnostic delay. Additionally, our study only included patient-reported symptoms and dates, which also could be a limitation. It would be a strength to have information from medical records in order to identify possible causes of diagnostic delay, but as we do not have a national register for clinical information on coeliac disease, this information was not available for this study. Therefore, there is still a need for more studies on the delay and diagnostic process of Danish coeliac disease patients, both survey studies and qualitative studies to identify potential barriers for timely diagnosis and reasons for diagnostic delay, for further progress in improving the diagnostic path for coeliac disease patients.

In conclusion, this study underlines the importance of the awareness of coeliac disease among all health care professionals, especially among the general practitioners who are the first point of contact with the health care system in Denmark. We found evidence of a significant diagnostic delay among Danish coeliac disease patients, both from the onset of symptoms and the first doctor's visit to diagnosis, but primarily due to the delay from the time of first health care contact to the time of diagnosis of coeliac disease.

## Figures and Tables

**Table 1:** Characteristics of the study-populations.

	Total study population *N* = 1,392	Delay study population *N* = 1,239
Age^†^ at survey, mean (SD)	42.8 years (21.1)	43.0 years (21.3)

Sex^‡^	*n* (% of 1,392)	*n* (% of 1,239)
Women	1,087 (78.1%)	971 (78.4%)
Men	303 (21.8%)	266 (21.5%)

Age at diagnosis^§^	*n* (% of 1,339^§^)	*n* (% of 1,200^§^)
0–9 years	226 (16.9%)	219 (18.3%)
10–19 years	174 (13.0%)	143 (11.9%)
20–39 years	399 (29.8%)	366 (30.5%)
40–59 years	426 (31.8%)	375 (31.3%)
60–79 years	114 (8.5%)	97 (8.1%)

Year of diagnosis	*n* (% of 1,382^¶^)	*n* (% of 1,239)
1945–1959	7 (0.5%)	6 (0.5%)
1960–1979	23 (1.7%)	23 (1.9%)
1980–1999	115 (8.3%)	113 (9.1%)
2000–2021	1,237 (88.9%)	1,097 (88.5%)

^†^45 participants missing information on age in total population, and 39 participants missing information on age in the delay population. ^‡^2 participants missing information on sex. ^§^Age in years calculated from year of birth and year of diagnosis. 53 missing age at diagnosis (45 missing year of birth and 10 missing year of diagnosis). For the delay population age calculated by year in differences from date of birth to date of diagnosis, 39 participants missing date of birth. ^¶^10 missing year of diagnosis in the total population.

**Table 2:** Answers from the questionnaire on health care follow-up visits, self-perceived health, evaluation of the diagnostic process, and burden of disease.

	*n* (% of 1,392)
Health care follow-up visits	
Visits during the last 12 months	898 (64%)
At the general practitioner^†^	309
At the hospital^†^	664
At a specialist in gastroenterology, private practice^†^	48
No follow-up	378 (27%)
Missing information on follow-up	116 (8%)

Self-perceived health	
Excellent	99 (7%)
Very good	434 (31%)
Good	591 (42%)
Fair	210 (15%)
Poor	46 (3%)
Do not know/wish not to say	12 (1%)

Satisfied with the diagnostic process?	
Very much	409 (29%)
Quite a lot	153 (11%)
Some	204 (15%)
A little	284 (20%)
Not at all	280 (20%)
Do not know/wish not to say	62 (4%)

Does the diagnosis of coeliac disease limit your daily life?	
Very much	114 (8%)
Quite a lot	229 (16%)
Some	603 (43%)
A little	370 (27%)
Not at all	74 (5%)
Do not know	2 (0.1%)

^†^Possible with more than one follow-up; 114 participants had follow-ups at more than one doctor.

**Table 3:** Self-reported symptoms before and/or at the time of diagnosis.

Symptoms^†^	*n* (% of total *N* (1,392))
Tiredness	948 (68%)
Abdominal pain	907 (65%)
Alternating stool	898 (64%)
Bloating	837 (60%)
Weight loss	501 (36%)
Anaemia	423 (30%)
Nausea	355 (26%)
Joint pain	342 (25%)
Headache	334 (24%)
Dizziness	202 (15%)
Failure to thrive (children)	195 (14%)
Osteoporosis	96 (7%)
Infertility	56 (4%)
Other symptoms than listed	261 (19%)
No symptoms	31 (2%)
Do not know	6 (0.4%)

^†^Possibility for several symptoms per participant.

**Table 4:** Self-reported diagnostic delay in a Danish population of patients with coeliac disease.

	Patients' diagnostic delay	Doctors' diagnostic delay	Total diagnostic delay
Mean diagnostic delay (SD)	1.8 years (5.0)	4.0 years (8.0)	5.8 years (9.5)
Diagnostic delay of more than 3 years (%)	14.5%	26.3%	37.9%
Diagnostic delay of more than 10 years (%)	5.3%	12.4%	18.6%

	Mean years (SD)	Mean years (SD)	Mean years (SD)

Year of diagnosis			
1945–1959 (*n* = 6)	0.1 (0.3)	0.3 (0.4)	0.5 (0.5)
1960–1979 (*n* = 23)	1.0 (2.6)	2.2 (4.7)	3.2 (5.6)
1980–1999 (*n* = 113)	1.9 (5.6)	5.7 (10.2)	7.6 (11.4)
2000–2021 (*n* = 1097)	1.8 (5.0)	3.8 (7.8)	5.7 (9.4)
Age at diagnosis for the 1097 participants diagnosed during the years 2000–2021^†^			
0–9 years (*n* = 191)	0.4 (1.0)	1.1 (1.5)	1.6 (1.8)
10–19 years (*n* = 134)	1.4 (2.8)	2.0 (3.3)	3.5 (4.1)
20–39 years (*n* = 311)	1.9 (4.2)	3.4 (6.3)	5.3 (7.3)
40–59 years (*n* = 330)	2.8 (7.4)	6.1 (10.3)	8.9 (12.6)
60–79 years (*n* = 97)	1.2 (3.2)	5.8 (12.2)	7.1 (12.7)

^†^34 participants missing age at diagnosis. SD: standard derivation.

## Data Availability

Restrictions apply to the availability of these data according to Danish law. Therefore, the data and the information regarding the participants cannot be publicly available. A request for access to the data needs approval from appropriate Danish authorities and are subject to Danish regulations on personal data protection. A request for arrangement of data transfer agreements can be sent to the corresponding author.

## Supplementary Materials

Supplementary Materials Supplemental Table S1: Self-reported symptoms before diagnosis among participants with and without a diagnostic delay of more than 10 years.
