# Supplementary material for: Diagnostic Delay in Coeliac Disease: A Survey among Danish Patients
Source: Can J Gastroenterol Hepatol. 2022 Dec 28;2022:5997624. doi: 10.1155/2022/5997624 (PMC9812619; doi:10.1155/2022/5997624)
Supplement: Supplementary Materials — Supplemental Table S1: Self-reported symptoms before diagnosis among participants with and without a diagnostic delay of more than 10 years. [file 5997624.f1.docx]

**Supporting Information**

***Diagnostic delay in coeliac disease: a survey among Danish patients***

Authors: Line Lund Kårhus^1^, Susanne Hansen^1^, Jüri J. Rumessen^1^, Allan Linneberg^1,2^

^1^ Center for Clinical Research and Prevention, Copenhagen University Hospital - Bispebjerg and Frederiksberg, Copenhagen, Denmark

^2^ Department of Clinical Medicine, Faculty of Health and Medical Sciences, University of Copenhagen, Copenhagen, Denmark

| **Supplemental Table S1**: Self-reported symptoms before diagnosis among participants with and without a diagnostic delay of more than 10 years | | |
| --- | --- | --- |
| Symptoms^†^ | Participants with more than 10 years of total diagnostic delay  n (% of 230) | Participants with less than 10 years of total diagnostic delay  n (% of 1,009) |
| Tiredness | 178 (77%) | 700 (69%) |
| Abdominal pain | 178 (77%) | 667 (66%) |
| Alternating stool | 169 (73%) | 667 (66%) |
| Bloating | 177 (77%) | 602 (60%) |
| Weight loss | 85 (37%) | 389 (39%) |
| Anaemia | 108 (47%) | 281 (28%) |
| Nausea | 69 (30%) | 275 (27%) |
| Joint pain | 99 (43%) | 226 (22%) |
| Headache | 71 (31%) | 240 (24%) |
| Dizziness | 46 (20%) | 144 (14%) |
| Failure to thrive (children) | 16 (7%) | 162 (16%) |
| Osteoporosis | 31 (13%) | 56 (6%) |
| Infertility | 25 (11%) | 24 (2%) |
| Other symptoms than listed | 58 (25%) | 173 (17%) |
| No symptoms | 1 (0.4%) | 5 (0.5%) |
| *^†^possibility for several symptoms per participant* | | |
